# Pulmonary emphysema and coronary artery calcifications at baseline LDCT and long-term mortality in smokers and former smokers of the ITALUNG screening trial

**DOI:** 10.1007/s00330-023-09504-4

**Published:** 2023-03-01

**Authors:** Mario Mascalchi, Chiara Romei, Chiara Marzi, Stefano Diciotti, Giulia Picozzi, Francesco Pistelli, Marco Zappa, Eugenio Paci, Francesca Carozzi, Giuseppe Gorini, Fabio Falaschi, Anna Lisa Deliperi, Gianna Camiciottoli, Laura Carrozzi, Donella Puliti

**Affiliations:** 1 Department of Clinical and Experimental, Biomedical Sciences “Mario Serio, ” University of Florence, Viale Pieraccini, 50134 Florence, Italy; 2 Division of Epidemiology and Clinical Governance, Institute for Study, PRevention and netwoRk in Oncology (ISPRO), Florence, Italy; 3 Division of Cancer Epidemiology (C020), German Cancer Research Center (DKFZ), Heidelberg, Germany; 4 Division of Radiology, Cisanello Hospital, Pisa, Italy; 5 “Nello Carrara” Institute of Applied Physics, National Research Council of Italy, Sesto Fiorentino, Florence, Italy; 6 Department of Electrical, Electronic, and Information Engineering ‘Guglielmo Marconi’, University of Bologna, Bologna, Italy; 7 Pulmonary Unit, Cardiothoracic and Vascular Department, Pisa University Hospital, Pisa, Italy; 8 Regional Laboratory of Cancer Prevention, Institute for Cancer Research, Prevention and Clinical Network (ISPRO), Florence, Italy

**Keywords:** Cardiovascular disease, Cause of death, Pulmonary emphysema, Lung neoplasm, Smokers

## Abstract

**Objectives:**

Cardiovascular disease (CVD), lung cancer (LC), and respiratory diseases are main causes of death in smokers and former smokers undergoing low-dose computed tomography (LDCT) for LC screening. We assessed whether quantification of pulmonary emphysematous changes at baseline LDCT has a predictive value concerning long-term mortality.

**Methods:**

In this longitudinal study, we assessed pulmonary emphysematous changes with densitometry (volume corrected relative area below − 950 Hounsfield units) and coronary artery calcifications (CAC) with a 0–3 visual scale in baseline LDCT of 524 participants in the ITALUNG trial and analyzed their association with mortality after 13.6 years of follow-up using conventional statistics and a machine learning approach.

**Results:**

Pulmonary emphysematous changes were present in 32.3% of subjects and were mild (6% ≤ RA950 ≤ 9%) in 14.9% and moderate-severe (RA950 > 9%) in 17.4%. CAC were present in 67% of subjects (mild in 34.7%, moderate-severe in 32.2%). In the follow-up, 81 (15.4%) subjects died (20 of LC, 28 of other cancers, 15 of CVD, 4 of respiratory disease, and 14 of other conditions). After adjusting for age, sex, smoking history, and CAC, moderate-severe emphysema was significantly associated with overall (OR 2.22; 95CI 1.34–3.70) and CVD (OR 3.66; 95CI 1.21–11.04) mortality. Machine learning showed that RA950 was the best single feature predictive of overall and CVD mortality.

**Conclusions:**

Moderate-severe pulmonary emphysematous changes are an independent predictor of long-term overall and CVD mortality in subjects participating in LC screening and should be incorporated in the post-test calculation of the individual mortality risk profile.

**Key Points:**

*• Densitometry allows quantification of pulmonary emphysematous changes in low-dose CT examinations for lung cancer screening.*

*• Emphysematous lung density changes are an independent predictor of long-term overall and cardio-vascular disease mortality in smokers and former smokers undergoing screening.*

*• Emphysematous changes quantification should be included in the post-test calculation of the individual mortality risk profile.*

**Supplementary Information:**

The online version contains supplementary material available at 10.1007/s00330-023-09504-4.

## Introduction

Cardio-vascular disease (CVD), lung cancer (LC), and respiratory diseases are the three main causes of death in smokers and former smokers undergoing low-dose computed tomography (LDCT) for LC screening [1–4]. The latter is currently recommended in the USA for subjects 50–80 years of age with a smoking history of at least 20 pack-years or who have quit in the past 15 years [5]. Notably, LDCT also enables visualization and assessment of smoking-related biomarkers of co-morbidities, in particular coronary artery calcifications (CAC) and pulmonary emphysematous changes [6]. Although the correlation between emphysematous changes on CT and clinical or functional evidence of airflow obstruction qualifying for chronic obstructive lung disease (COPD) is weak [7], there is growing interest in incorporating post-LDCT information concerning potential biomarkers of smoking-related co-morbidities as CAC and emphysematous changes for the definition of the individual risk profile [8–10]. This might improve LC risk stratification, adapt screening LDCT intervention (starting age, frequency of LDCT rounds, and stopping age), and predict individual mortality outcome for smoking-related competing causes [11], according to a personalized medicine approach which maximizes the benefit and minimizes the harms of LC screening.

Assessment of pulmonary emphysematous changes in LDCT examinations for LC screening can be performed visually [12, 13] or with densitometry [11, 14, 15]. Lung densitometry is less affected by inter-observer variability [16, 17] and better allows stratification for emphysema severity [18].

In this study, we assessed the association between quantification of emphysematous changes at baseline LDCT and mortality after 13 years since randomization in subjects recruited in the ITALUNG randomized clinical trial [2].

## Materials and methods

### Participants’ selection and characterization

The ITALUNG trial (clinical trial registration, number = NCT02777996) was approved by the Local Ethics Committee of the three participating institutions in the Tuscany region of Italy (approval number 29–30 of 30 September 2003; number 23 of 27 October 2003; and number 00028543 of 13 May 2004), and each participating subject provided written informed consent. Protocols, LDCT results, and mortality were previously reported [2, 19, 20]. Eligible were 55–69 years aged subjects with a smoking history ≥ 20 pack-years and who were current smokers or have quitted in the last 10 years. Overall, 1613 subjects were randomized to receive 4 annual LDCT and 1593 to usual care. Baseline LDCT was obtained between 2004 and 2006 in 1406 subjects of the active arm. In ITALUNG, we employed 8 different spiral CT scanners from two vendors over the 2004–2012 period of LDCT active screening [21]. CT scanner technology affects the quantification of emphysematous changes because it defines the range of acquisition parameters among which slice collimation and image reconstruction filtering are the most important [17, 22]. In 536 subjects recruited in two (Florence and Pisa) screening centers of ITALUNG, the baseline LDCT examination was obtained using two identical 4 rows of detector CT scanners (Somatom Volume Zoom, Siemens Healthcare) and the same LDCT examination protocol. This baseline LDCT constituted the basis for the present investigation.

### LDCT and its evaluation

The scanners were calibrated on air daily. The acquisition technique for LC screening included 1-mm collimation slices from the apex to the base, beam pitch 1.75 × 4, reconstruction 1.25 mm, 140 kV, and 40 mAs, during one breath-hold obtained at end-inspiration.

Before the assessment, the quality of LDCT examination images was judged as adequate or inadequate due to artifacts due to motion, metallic stent placement, etc.

#### Emphysema

We performed a quantitative assessment of lung emphysematous changes with densitometry in images reconstructed with the smooth (b31f Siemens) kernel. For such a purpose, we used the Pulmo (Siemens, Healthcare) software in the Florence cohort and the SYNAPSE 3D (Fujifilm) software in the Pisa cohort, according to an established procedure [17]. The presence of emphysematous changes was defined using the 6.0% threshold of the relative area of the lung with density values below 950 Hounsfield units (RA950) [18] normalized to the lung volume at acquisition. Subjects with emphysematous changes were then arbitrarily divided into those with mild (6% ≤ RA950 ≤ 9%) and those with moderate-severe (RA950 > 9%) emphysema.

#### CAC

CAC visualized in LDCT for LC screening are established predictors of overall and CVD mortality in the screened population [6, 23, 24]. Hence, they must be assessed in order to control for this possible confounding variable. Accordingly, in the present study, we evaluated CAC with a visual semi-quantitative scoring system which is fast, reproducible, and performed as well as the Agatston score in predicting CV events and mortality [23] and whose results in the whole ITALUNG cohort were previously reported [25]. By evaluating the entire coronary arterial vessels, it distinguishes 4 scores: 0 = absent, 1 = mild (isolated flecks of CAC within a segment), 2 = moderate (any degree between mild and severe), and 3 = severe (continuous CAC within a segment). Here, we considered moderate and severe CAC altogether, since they significantly predict CVD mortality as opposed to absent or mild CAC [25].

Assessment of both pulmonary emphysema and CAC was possible in baseline LDCT of 524 subjects (267 subjects of the Pisa cohort and 257 subjects of the Florence cohort), whereas 12 subjects were excluded because of technically inadequate baseline LDCT. The characteristics of the 524 subjects who were finally enrolled are reported in Table 1. Notably, their age, gender, smoking history, and CAC severity were similar to those in the remainder of 840 subjects undergoing baseline LDCT in ITALUNG for whom the CAC evaluation was available (Table 1).

**Table 1:** Characteristics at baseline LDCT of 524 subjects included in the study and in 840 subjects belonging to the active arm of ITALUNG for whom the coronary artery calcifications (CAC) visual evaluation was possible

	Study (*n* = 524)		Others (*n* = 840)		
	No. of subjects	%	No. of subjects	%	*p* value
Demographic characteristics					
Gender					
Male	355	67.7%	535	63.7%	
Female	169	32.3%	305	36.3%	*p* = 0.126
Age at randomization					
< 60	265	50.6%	381	45.4%	
60–70	150	28.6%	259	30.8%	
65–70	109	20.8%	200	23.8%	*p* = 0.160
Smoking history					
Smoking status					
Current	345	65.8%	555	66.1%	
Former	179	34.2%	285	33.9%	*p* = 0.930
Pack-years					
20–30	108	20.6%	197	23.5%	
30–40	154	29.4%	204	24.3%	
40–50	127	24.2%	218	26.0%	
> 50	135	25.8%	221	26.3%	*p* = 0.189
Emphysema (RA950)					
No (< 6%)	355	67.8%	*Data not known*		
Mild (6–9%)	78	14.9%	*Data not known*		
Moderate/severe (> 9%)	91	17.4%	*Data not known*		
CAC visual score					
No	173	33.0%	297	35.4%	
Mild	182	34.7%	251	29.9%	
Moderate/severe	169	32.3%	292	34.8%	*p* = 0.173

### Follow-up

The 524 subjects (and the remainder of the ITALUNG active group) were followed up until 31 December 2018 with a median follow-up time from the date of randomization of 13.6 years (Q1–Q3: 12.9–13.9 years). The incidence of LC in the cohort was established by a link to the tumor registry of the Tuscany region. Follow-up for vital status and cause of death was performed through the linkage with the regional mortality registry and included a check for residential status. Causes of death were coded according to the International Classification of Disease (ICD) version IX for subjects deceased until 2009 and version X for subjects deceased from 2010 onwards considering LC (ICD-9 code 162 and ICD-10 code C33-C34), other cancers (ICD-9 codes 140–239 and ICD-10 codes C00-D48), CVD (ICD-9 codes 390–459 and ICD-10 codes I00-I99), and respiratory illness (ICD-9 codes 460–519 and ICD-10 codes J00-J99).

### Statistical analyses

Person-years at risk were calculated from the date of randomization either to the date of death or the date of censoring (migration or end of follow-up). Comparisons of frequency distribution between categories were performed using the chi-square test. Adjusted mortality ratio and confidence interval were estimated using the Cox model. All data analyses were conducted using Stata software, version 16.1.

### Machine learning analysis

Based on the results of conventional statistics (see below), we used machine learning techniques to evaluate the contribution to the prediction of the overall, LC, and CVD mortality of the following features: age, gender, smoking status, pack-years, CAC visual score, and RA950. To this aim, we trained, validated, and tested the eXtreme Gradient Boosting (XGBoost) model (with default parameters) [26] that is a tree-based machine learning model widely used to achieve cutting-edge performance on various recent machine learning challenges. We adopted a repeated (ten times) stratified tenfold cross-validation strategy for LC and CVD mortality prediction. For the overall mortality prediction, given the larger number of subjects in each class (i.e., 81 subjects had died as of December 31, 2018, vs. 443 who did not), we performed a repeated (ten times) stratified nested 10–fivefold (i.e., outer tenfold and inner fivefold) cross-validation, to estimate the unbiased generalization performance of the model along with performing, at the same time, hyperparameter optimization [27]. The hyperparameter space was defined as follows: the step size shrinkage used in the update to prevent overfitting, i.e., *eta* ∈ (0.5, 0.7, 0.9), the minimum loss reduction required to make a further partition on a leaf of the tree, i.e., *gamma* ∈ (10, 100, 1000), and the maximum depth of a tree, i.e., *max_depth* ∈ (2, 3, 4, 5). The performance was measured through the area under the receiver operating characteristic curve (AUROC), and we computed the average AUROC over the ten repetitions. Then, we interpreted predictions by using the Shapley additive explanations (SHAP) approach [28]. By assigning each feature an importance value for each sample, SHAP is a powerful explainable artificial intelligence (XAI) framework for evaluating predictions based on traditional Shapley values from game theory [29]. This method may explain the model locally (on a single sample) and globally. All machine learning analyses were performed using in-house Python code (Version 3.7.0) on an M1 MacBook Air (macOS Monterey version 12.6).

## Results

Pulmonary emphysematous changes were present in 169 (32.3%) of the 524 subjects enrolled in the study and were mild in 14.9% and moderate-severe in 17.4% (Table 1). The majority (351/524; 67%) of subjects showed some degree of CAC which were mild in 34.7% and moderate or severe in 32.2%. Notably, age, gender, smoking history, and CAC severity in the 524 subjects were similar to those in the remainder 840 subjects recruited in ITALUNG for whom CAC could be visually assessed (see Table 1). The presence of emphysematous changes (any degree) was correlated with male sex, age, and status of former smokers, whereas the presence of CAC (any degree) was correlated with male sex, age, and pack-years (Table 2). There was no correlation between the presence or severity of pulmonary emphysematous changes and CAC (Table 2).

**Table 2:** Correlation analysis between demographic and smoking history variables and pulmonary emphysematous changes defined as lung relative area below 950 Hounsfield units (RA950) ≥ 6% and coronary artery calcification (CAC) at baseline LDCT in 524 subjects

	Subjects with emphysema (RA950 ≥ 6%)		Subjects with CAC (any degree)	
	%	*p* value	%	*p* value
Gender				
Male *n* = 355	39.2% (*n* = 139/355)		71.8% (*n* = 255/355)	
Female *n* = 169	17.8% (*n* = 30/169)	*p* < 0.001	56.8% (*n* = 96/169)	*p* = 0.001
Age (years) at randomization				
< 60 *n* = 265	26.4% (*n* = 70/265)		55.1% (*n* = 146/265)	
60–70 *n* = 150	32.7% (*n* = 49/150)		76.7% (*n* = 115/150)	
65–70 *n* = 109	45.9% (*n* = 50/109)	*p* = 0.001	82.6% (*n* = 90/109)	*p* < 0.001
Smoking status				
Current *n* = 345	23.8% (*n* = 82/345)		65.8% (*n* = 227/345)	
Former *n* = 179	48.6% (*n* = 87/179)	*p* < 0.001	69.3% (*n* = 124/179)	*p* = 0.422
Pack-years				
20–30 *n* = 108	26.9% (*n* = 29/108)		63.9% (*n* = 69/108)	
30–40 *n* = 154	32.5% (*n* = 50/154)		57.8% (*n* = 89/154)	
40–50 *n* = 127	30.7% (*n* = 39/127)		68.5% (*n* = 87/127)	
> 50 *n* = 135	37.8% (*n* = 51/135)	*p* = 0.325	78.5% (*n* = 106/135)	*p* = 0.002
Emphysema (RA950)				
No (< 6%) *n* = 355			66.5% (*n* = 236/355)	
Mild (6–9%) *n* = 78			61.5% (*n* = 48/78)	
Moderate/severe (> 9%) *n* = 91			73.6% (*n* = 67/91)	*p* = 0.234
CAC visual score				
None *n* = 173	31.2% (*n* = 54/173)			
Mild *n* = 182	31.9% (*n* = 58/182)			
Moderate/severe *n* = 169	33.7% (*n* = 57/169)	*p* = 0.875		

No difference in LC incidence was observed in association with pulmonary emphysema or CAC (Supplementary E-Table 1).

In the follow-up period, 81 (15.4%) of 524 subjects died. Mortality and causes of death were similar in the remainder of the 840 subjects recruited in ITALUNG (Supplementary E-Table 2). Causes of death in the 81 subjects of the study group were LC in 20 subjects, other cancers in 28 subjects, CVD in 15 subjects, respiratory diseases in 4 subjects, and others in 14 subjects. The CVD mortality causes included ischemic heart disease (*n* = 8), atrial fibrillation (*n* = 2) intracerebral hemorrhage (*n* = 2), ruptured aneurysm of the abdominal aorta (*n* = 1), and other unspecified cardiovascular (*n* = 1) or cerebrovascular (*n* = 1). The respiratory mortality causes included interstitial pulmonary fibrosis (*n* = 2), COPD (*n* = 1), and unspecified respiratory failure (*n* = 1).

In the study group, the overall mortality rate ratios significantly increased with a moderate-severe degree of emphysematous changes [OR 2.22 (95%CI 1.34–3.70)] after adjusting for age, sex, smoking history, screening center, and CAC visual score. Moderate-severe degree of CAC was associated with an increased overall mortality, but the difference was not statistically significant [OR 1.51 (95%CI 0.94–2.43)] (Table 3). The adjusted CVD mortality rate ratios significantly increased with a moderate-severe degree of emphysematous changes [OR 3.66 (95%CI 1.21–11.04)] and with a moderate-severe degree of CAC [OR 3.18 (95%CI 0.99–10.16)], while the adjusted LC mortality did not (Table 3).

**Table 3:** Overall, lung cancer and cardio-vascular mortality after a median follow-up of 13.6 years (Q1–Q3: 12.9–13.9) since randomization in 524 subjects

Overall mortality	Person years	Deaths	Mortality rate × 1000	Crude mortality rate ratio (95%CI)	Adjusted* mortality rate ratio (95%CI)
Emphysema (RA950)					
No/mild (< 9%)	5629.8	56	9.95	Ref	Ref
Moderate/severe (> 9%)	1127.2	25	22.18	2.35 (1.46–3.76)	2.23 (1.34–3.70)
CAC visual score					
No/mild	4645.9	41	8.82	Ref	Ref
Moderate/severe	2111.1	40	18.95	2.24 (1.45–3.47)	1.51 (0.94–2.43)
Lung cancer mortality	Person years	LC deaths	Mortality rate × 1000	Crude mortality rate ratio (95%CI)	Adjusted* mortality rate ratio (95%CI)
Emphysema (RA950)					
No/mild (< 9%)	5629.8	16	2.84	Ref	Ref
Moderate/severe (> 9%)	1127.2	4	3.55	1.32 (0.44–3.94)	1.16 (0.36–3.76)
CAC visual score					
No/mild	4645.9	12	2.58	Ref	Ref
Moderate/severe	2111.1	8	3.79	1.59 (0.65–3.90)	0.94 (0.36–2.47)
Cardio-vascular mortality	Person years	CV deaths	Mortality rate × 1000	Crude mortality rate ratio (95%CI)	Adjusted* mortality rate ratio (95%CI)
Emphysema (RA950)					
No/mild (< 9%)	5629.8	9	1.60	Ref	Ref
Moderate/severe (> 9%)	1127.2	6	5.32	3.75 (1.33–10.60)	3.66 (1.21–11.04)
CAC visual score					
No/mild	4645.9	6	1.29	Ref	Ref
Moderate/severe	2111.1	9	4.26	3.53 (1.25–9.92)	3.18 (0.99–10.16)

^*^ Adjusted for age, sex, smoking history, screening center, emphysema and CAC visual score

**Fig. 1:**
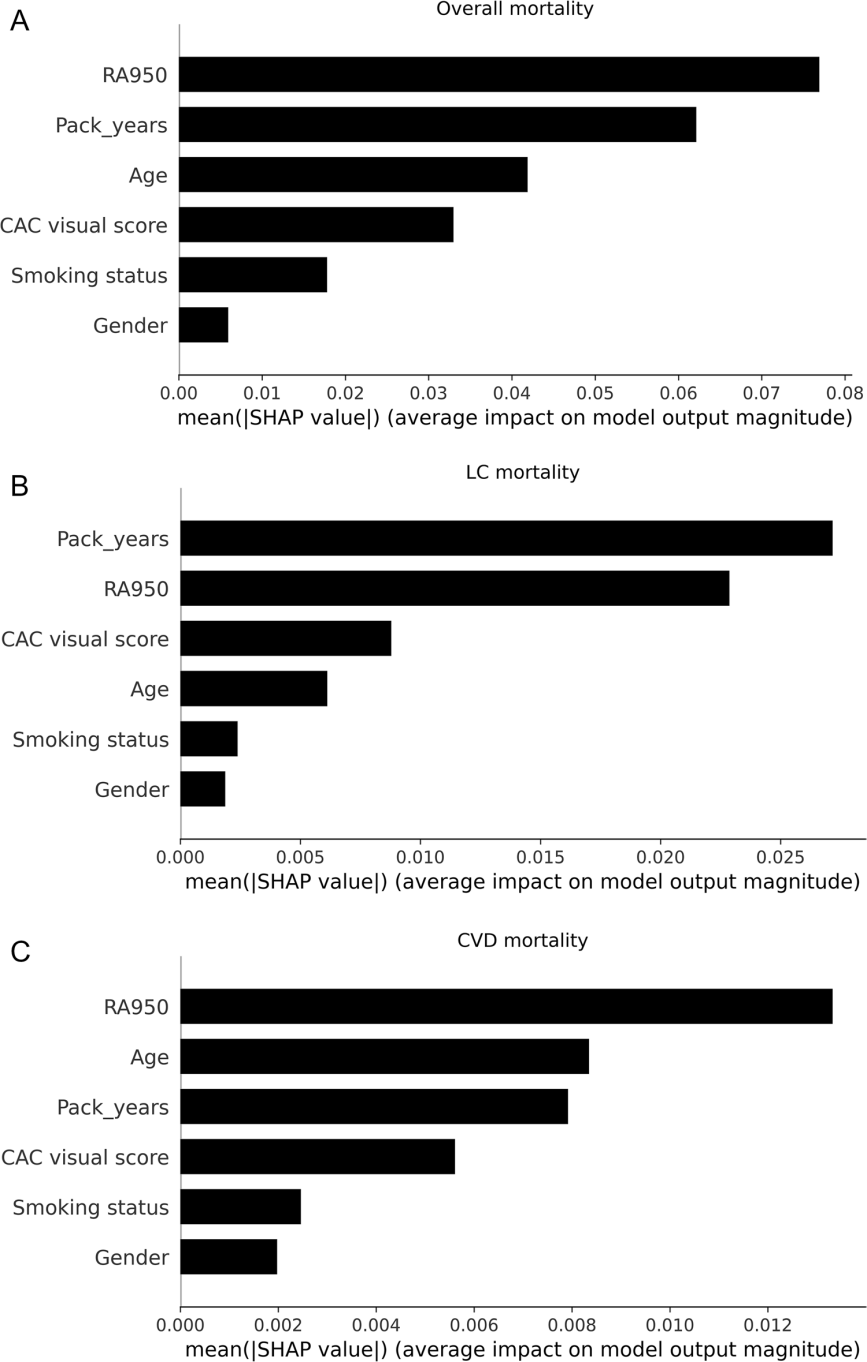
Summary bar plot showing the global relevance of each feature, as measured with the mean absolute SHAP value. Mean absolute SHAP values have been reported for the overall mortality (**A**), LC mortality (**B**), and CVD mortality (**C**)

Machine learning analysis (Fig. 1) showed that RA950 was the best single feature for the prediction of overall and CVD mortality among age, gender, pack-years, smoking status, and CAC visual score, with AUROC values of 0.70 for overall and 0.73 for CVD mortality. For the prediction of LC mortality, the two most important features were pack-years and RA950, with AUROC = 0.61.

## Discussion

In the present investigation, we quantitatively assessed pulmonary emphysematous changes in baseline LDCT of one sub-cohort of subjects recruited in the ITALUNG trial and analyzed 13 years after randomization of its potential impact on death and its causes, while controlling for demographic and smoking history variables, as well as CAC that are relevant predictors of overall and CVD mortality in the LC screening population [23–25]. Our data, as assessed with both conventional statistics and a machine learning approach, indicate that quantification of emphysematous changes at baseline LDCT independently predicts overall and CVD mortality in heavy smokers and former smokers undergoing LC screening.

Our sample was selected based on the type of CT scanner and acquisition protocol which substantially affect the quantitative assessment of diffuse lung changes [17, 18], but was representative of the entire group undergoing LDCT screening in ITALUNG. Also, the prevalence and distribution of pulmonary emphysematous changes [6, 14] and CAC [6, 23–25] in the sub-cohort were substantially consistent with previous data in subjects undergoing LC screening. So far, quantitative evaluation of pulmonary emphysematous changes in subjects undergoing screening LDCT has been correlated with the incidence of LC [15] and with the Pulmonary Functional Test (PFT) results [30, 31]. Moreover, emphysematous changes were significantly correlated with the risk of a next CV event [32], and the addition of mean lung density and RA950 lung percentage improved the prediction of LC incidence and especially of both COPD and CVD mortality in the National Lung Screening Trial (NLST) [11]. As a matter of fact, CVD is the first cause of death in patients with COPD exceeding respiratory failure and LC [33–35] consistently with the view that COPD is a powerful, independent risk factor for CV morbidity and mortality. The predictive value of emphysematous changes in baseline LDCT with respect to overall and CVD mortality in our study is in line with the above evidence. In general, since smoke is a risk factor for both COPD and vascular damage, one may assume that the correlation between emphysematous changes and CVD mortality could be mediated by CAC, and it is established that CAC are more frequent in subjects with COPD [36, 37]. However, our data indicate that the association between emphysematous changes and CVD mortality is independent from that between CAC and CVD mortality. This is not surprising because the mechanisms underlying the association of emphysematous changes or COPD with CVD are not entirely accounted for by those of the association between CAC and CVD. In fact, several additional mechanisms have been identified. Systemic inflammation in response to smoking and air or professional pollution is implicated in both lung parenchymal disruption and airway remodelling and the genesis and evolution of atherosclerotic plaque [38]. In particular, in COPD patients with emphysema, also osteoporosis can contribute to a low-grade systemic inflammation. Osteoporosis is highly prevalent in these patients [39, 40] for the decreased physical activity and the frequently administered steroid therapy, and is associated with increased plasma levels of C-reactive protein, interleukin 6, and tumor necrosis factor alpha that further increase during exacerbations of lung disease. A second link between emphysematous changes and COPD and CVD can be represented by sustained or intermittent hypoxia due to gas exchange derangement creating a vicious circle of hypoxia and re-oxygenation. Overall, the relationship between pulmonary emphysematous changes and CVD mortality could reflect a dynamic multi-faceted interaction between lung and vessels with a possible role for inflammation, endothelial and elastin deficit, and hypoxia due to gas exchange derangements, while arterial calcium deposition can be considered a more advanced expression of arterial wall damage. Certainly, future researches are worthy on the interplays among pulmonary emphysema, COPD, CAC, and CVD risk and mortality in subjects with significant smoking history. Notably, in our study, the association between emphysematous changes and LC mortality was weak, but this could reflect the fact that screening typically detects and allows treatment and cure of early-stage LC.

Despite the above physio-pathological uncertainties, our data, confirming that emphysematous changes in baseline screening LDCT significantly and independently predict overall and CVD mortality [11], suggest that this biomarker of co-morbidity should be considered in post-test individual risk stratification [8–10].

We recognize the following limitations of our study. First, due to CT scanner heterogeneity, our data were obtained in a part only (37.2%, 524/1406), although representative, of the subjects who received baseline LDCT in the ITALUNG trial. Investigation in larger samples, as well as external validation of the machine learning algorithm in independent samples, is worthy. In particular, the low number of deaths due to respiratory diseases, which is in line with other studies [4, 41], did not allow us to assess the predictive value of emphysematous changes with respect to respiratory causes of death that have been observed in the NLST trial [11, 13]. Second, although measurement of airway thickness is feasible in LDCT for LC screening [11, 42], we did not perform it. This may have obscured the contribution of the full spectrum of the changes underlying COPD which can be assessed on CT to the mortality profile of our subjects. Third, we used two software for emphysema quantification which were applied to images acquired with the same CT scanners and protocols. However, in a study comparing the results of 8 software for emphysema assessment in 50 patients, the inter-software mean bias was within ± 0.46%, for RA950 [43]. Fourth, at the time of baseline screening in ITALUNG (2004–2006), no clear implications for CAC or emphysematous changes detected in LDCT examinations were established. Accordingly, although it was possible that visual evidence of moderate-severe CAC or emphysematous changes was sporadically contained in the screening test report provided to the subject and his/her general practitioner, in the present retrospective study, we could not assess the implications and consequences of such a report. Certainly, as demonstrated by our study, these biomarkers of smoking-related co-morbidities can be assessed on baseline LDCT, are no longer to be ignored, and deserve consideration in prospective LDCT screening cohorts. Fifth, visual assessment allows detailed assessment of the distribution and types of lung emphysematous changes, including centrilobular, panlobular, and paraseptal [18], which we did not analyze. The possibility that these additional features may better explain the mortality implications of the emphysematous changes in baseline LDCT of people undergoing LC screening might deserve additional studies.

## Conclusions

Pulmonary emphysematous changes at baseline LDCT is independently associated with long-term overall and CVD death in subjects recruited for LC screening and should be incorporated in the post-test calculation of the individual mortality risk profile.

## Supplementary Information

Below is the link to the electronic supplementary material.

Supplementary file1 (PDF 120 KB)

## Data Availability

The data of this study are available on request at Professor Mario Mascalchi at mario.mascalchi@unifi.it.

## Abbreviations

AUROC: Area under the receiver operating characteristic curve
CAC: Coronary artery calcifications
COPD: Chronic obstructive pulmonary disease
CT: Computed tomography
CVD: Cardio-vascular disease
ICD: International Classification of Disease
LC: Lung cancer
LDCT: Low-dose computed tomography
NLST: National Lung Screening Trial
OR: Odds ratio
PFT: Pulmonary Functional Test
RA950: Relative area of the lung with density values below 950 Hounsfield units
SHAP: Shapley additive explanations
XAI: EXplainable Artificial Intelligence framework

## Declarations

### Guarantor

The scientific guarantor of this publication is Mario Mascalchi.

### Statistics and biometry

Donella Puliti, a senior statistician at ISPRO, performed the statistical analysis of the data for this manuscript.

### Study subjects or cohorts overlap

Some study subjects or cohorts have been previously reported in Lopes-Pegna et al Lung Cancer Lung Cancer (2009) 64:34–40; Camiciottoli et al Eur Radiol (2009)19:58–66; Paci et al Thorax (2017) 72:825–831; Mascalchi et al Eur J Radiol (2021) 10.1016/j.ejrad.2021.110040.

### Methodology

• prospective

• case–control study

• multicenter study
