# Supplementary material for: Pulmonary emphysema and coronary artery calcifications at baseline LDCT and long-term mortality in smokers and former smokers of the ITALUNG screening trial
Source: Eur Radiol. 2023 Mar 1;33(5):3115–23. doi: 10.1007/s00330-023-09504-4 (PMC10121526; doi:10.1007/s00330-023-09504-4)
Supplement: Supplementary file 1 — Supplementary file1 (PDF 120 KB) [file 330_2023_9504_MOESM1_ESM.pdf]

Supplementary table 1. Lung cancer incidence, pulmonary emphysematous changes and coronary artery calcifications at baseline LDCT in 524 subjects.

**Emphysema (RA950)**	Lung cancers	LC incidence rate x1000	Crude LC incidence rate ratio (95%CI)	Adjusted* LC incidence rate ratio (95%CI)
No/Mild (≤9%)	21	4.38	ref	ref
Moderate/severe (>9%)	4	4.06	0.93 (0.32 - 2.72)	1.01 (0.33 - 3.11)
**CAC visual score**				
No/Mild	16	4.04	ref	ref
Moderate/severe	9	4.94	1.23 (0.54 - 2.78)	0.86 (0.36 - 2.07)

* Adjusted for age, sex, smoking history, screening center, emphysema, and CAC visual score.

Supplementary Table 2. Mortality and causes of death in ITALUNG

	**Study (n=524)**		**Others (n=840)**		**p-value**
	Lung cancers	LC incidence rate x1000	Lung cancers	LC incidence rate x1000	
Overall	25	4.32	57	6.40	p=0.107
	Deaths	Mortality rate x1000	Deaths	Mortality rate x1000	
Overall	81	11.99	127	12.08	p=0.877
Lung cancer	20	2.96	42	4.00	p=0.263
Cardiovascular disease	15	2.22	20	1.90	p=0.742
